# Supplementary material for: Placental Pathology and Placental Growth Factor (PlGF)/Vascular Endothelial Growth Factor Receptor-1 (VEGFR-1) Pathway Expression Evaluation in Fetal Congenital Heart Defects
Source: Life (Basel). 2025 May 22;15(6):837. doi: 10.3390/life15060837 (PMC12194308; doi:10.3390/life15060837)
Supplement: Supplementary file 1 [file life-15-00837-s001.zip › life-3620360-supplementary.pdf]

Supplementary Materials

Table S1. Comparative evaluation of placental immunostaining intensities at the level of syncytiotrophoblast, decidual cells and villous endothelial cells for placental growth factor (PlGF).

**Staining intensity**	**Syncytiotrophoblast**	**Decidual cells**	**p-value ***
Absent staining, *n*	2 / 40	4 / 40	0.0748
Weak staining, *n*	23 / 40	29 / 40	
Moderate staining, *n*	10 / 40	4 / 40	
Strong staining, *n*	5 / 40	3 / 40	

**Staining intensity**	**Syncytiotrophoblast**	**Villous endothelial cells**	**p-value ***
Absent staining, *n*	2 / 40	18 / 40	<0.0001
Weak staining, *n*	23 / 40	18 / 40	
Moderate staining, *n*	10 / 40	3 / 40	
Strong staining, *n*	5 / 40	1 / 40	

**Staining intensity**	**Decidual cells**	**Villous endothelial cells**	**p-value ***
Absent staining, *n*	4 / 40	18 / 40	0.0046
Weak staining, *n*	29 / 40	18 / 40	
Moderate staining, *n*	4 / 40	3 / 40	
Strong staining, *n*	3 / 40	1 / 40	

* Cochran–Armitage test for trend.

Table S2. Comparative evaluation of placental immunostaining intensities at the level of syncytiotrophoblast, decidual cells and villous endothelial cells for vascular endothelial growth factor receptor-1 (VEGFR-1).

**Staining intensity**	**Syncytiotrophoblast**	**Decidual cells**	**p-value ***
Absent staining, *n*	1 / 40	1 / 40	0.4253
Weak staining, *n*	13 / 40	10 / 40	
Moderate staining, *n*	15 / 40	15 / 40	
Strong staining, *n*	11 / 40	14 / 40	
**Staining intensity**	**Syncytiotrophoblast**	**Villous endothelial cells**	**p-value ***
Absent staining, *n*	1 / 40	6 / 40	<0.0001
Weak staining, *n*	13 / 40	23 / 40	
Moderate staining, *n*	15 / 40	10 / 40	
Strong staining, *n*	11 / 40	1 / 40	
**Staining intensity**	**Decidual cells**	**Villous endothelial cells**	**p-value ***
Absent staining, *n*	1 / 40	6 / 40	<0.0001
Weak staining, *n*	10 / 40	23 / 40	
Moderate staining, *n*	15 / 40	10 / 40	
Strong staining, *n*	14 / 40	1 / 40	

* Cochran–Armitage test for trend.

Table S3. Comparative evaluation of placental immunostaining intensities of placental growth factor (PlGF) and vascular endothelial growth factor receptor-1 (VEGFR-1) in atrioventricular septal defect (AVSD) cases and controls.

Localization	Staining intensity	AVSD	Control	p-value *
**a. Placental growth factor (PlGF)**				
Syncytiotrophoblast	Absent staining, *n*	0 / 7	0 / 20	0.0435
	Weak staining, *n*	6 / 7	8 / 20	
	Moderate staining, *n*	1 / 7	8 / 20	
	Strong staining, *n*	0 / 7	4 / 20	
Decidual cells	Absent staining, *n*	2 / 7	0 / 20	0.0283
	Weak staining, *n*	5 / 7	14 / 20	
	Moderate staining, *n*	0 / 7	3 / 20	
	Strong staining, *n*	0 / 7	3 / 20	
Villous endothelial cells	Absent staining, *n*	4 / 7	8 / 20	0.3260
	Weak staining, *n*	3 / 7	10 / 20	
	Moderate staining, *n*	0 / 7	2 / 20	
	Strong staining, *n*	0 / 7	0 / 20	
**b. Vascular endothelial growth factor receptor-1 (VEGFR-1)**				
Syncytiotrophoblast	Absent staining, *n*	0 / 7	1 / 20	0.9841
	Weak staining, *n*	3 / 7	5 / 20	
	Moderate staining, *n*	2 / 7	10 / 20	
	Strong staining, *n*	2 / 7	4 / 20	
Decidual cells	Absent staining, *n*	1 / 7	0 / 20	0.3568
	Weak staining, *n*	2 / 7	3 / 20	
	Moderate staining, *n*	1 / 7	10 / 20	
	Strong staining, *n*	3 / 7	7 / 20	
Villus endothelial cells	Absent staining, *n*	1 / 7	3 / 20	0.6245
	Weak staining, *n*	5 / 7	12 / 20	
	Moderate staining, *n*	1 / 7	4 / 20	
	Strong staining, *n*	0 / 7	1 / 20	

AVSD, atrioventricular septal defect. * Cochran–Armitage test for trend.

Table S4. Comparative evaluation of placental immunostaining intensities of placental growth factor (PlGF) and vascular endothelial growth factor receptor-1 (VEGFR-1) in ventricular septal defect (VSD) cases and controls.

Localization	Staining intensity	VSD	Control	p-value *
**a. Placental growth factor (PlGF)**				
Syncytiotrophoblast	Absent staining, *n*	2 / 7	0 / 20	0.0035
	Weak staining, *n*	5 / 7	8 / 20	
	Moderate staining, *n*	0 / 7	8 / 20	
	Strong staining, *n*	0 / 7	4 / 20	
Decidual cells	Absent staining, *n*	1 / 7	0 / 20	0.0622
	Weak staining, *n*	6 / 7	14 / 20	
	Moderate staining, *n*	0 / 7	3 / 20	
	Strong staining, *n*	0 / 7	3 / 20	
Villous endothelial cells	Absent staining, *n*	4 / 7	8 / 20	0.9665
	Weak staining, *n*	2 / 7	10 / 20	
	Moderate staining, *n*	0 / 7	2 / 20	
	Strong staining, *n*	1 / 7	0 / 20	
**b. Vascular endothelial growth factor receptor-1 (VEGFR-1)**				
Syncytiotrophoblast	Absent staining, *n*	0 / 7	1 / 20	0.6868
	Weak staining, *n*	3 / 7	5 / 20	
	Moderate staining, *n*	1 / 7	10 / 20	
	Strong staining, *n*	3 / 7	4 / 20	
Decidual cells	Absent staining, *n*	0 / 7	0 / 20	0.2985
	Weak staining, *n*	3 / 7	3 / 20	
	Moderate staining, *n*	2 / 7	10 / 20	
	Strong staining, *n*	2 / 7	7 / 20	
Villus endothelial cells	Absent staining, *n*	1 / 7	3 / 20	0.9819
	Weak staining, *n*	4 / 7	12 / 20	
	Moderate staining, *n*	2 / 7	4 / 20	
	Strong staining, *n*	0 / 7	1 / 20	

VSD, ventricular septal defect. * Cochran–Armitage test for trend.

Table S5. Comparative evaluation of placental immunostaining intensities of placental growth factor (PlGF) and vascular endothelial growth factor receptor-1 (VEGFR-1) in hypoplastic left heart syndrome (HLHS) cases and controls.

Localization	Staining intensity	HLHS	Control	p-value *
**a. Placental growth factor (PlGF)**				
Syncytiotrophoblast	Absent staining, *n*	0 / 6	0 / 20	0.4071
	Weak staining, *n*	4 / 6	8 / 20	
	Moderate staining, *n*	1 / 6	8 / 20	
	Strong staining, *n*	1 / 6	4 / 20	
Decidual cells	Absent staining, *n*	1 / 6	0 / 20	0.1945
	Weak staining, *n*	4 / 6	14 / 20	
	Moderate staining, *n*	1 / 6	3 / 20	
	Strong staining, *n*	0 / 6	3 / 20	
Villous endothelial cells	Absent staining, *n*	2 / 6	8 / 20	0.6675
	Weak staining, *n*	3 / 6	10 / 20	
	Moderate staining, *n*	1 / 6	2 / 20	
	Strong staining, *n*	0 / 6	0 / 20	
**b. Vascular endothelial growth factor receptor-1 (VEGFR-1)**				
Syncytiotrophoblast	Absent staining, *n*	0 / 6	1 / 20	0.6929
	Weak staining, *n*	2 / 6	5 / 20	
	Moderate staining, *n*	2 / 6	10 / 20	
	Strong staining, *n*	2 / 6	4 / 20	
Decidual cells	Absent staining, *n*	0 / 6	0 / 20	0.5570
	Weak staining, *n*	2 / 6	3 / 20	
	Moderate staining, *n*	2 / 6	10 / 20	
	Strong staining, *n*	2 / 6	7 / 20	
Villus endothelial cells	Absent staining, *n*	1 / 6	3 / 20	0.5991
	Weak staining, *n*	2 / 6	12 / 20	
	Moderate staining, *n*	3 / 6	4 / 20	
	Strong staining, *n*	0 / 6	1 / 20	

HLHS, hypoplastic left heart syndrome. * Cochran–Armitage test for trend.

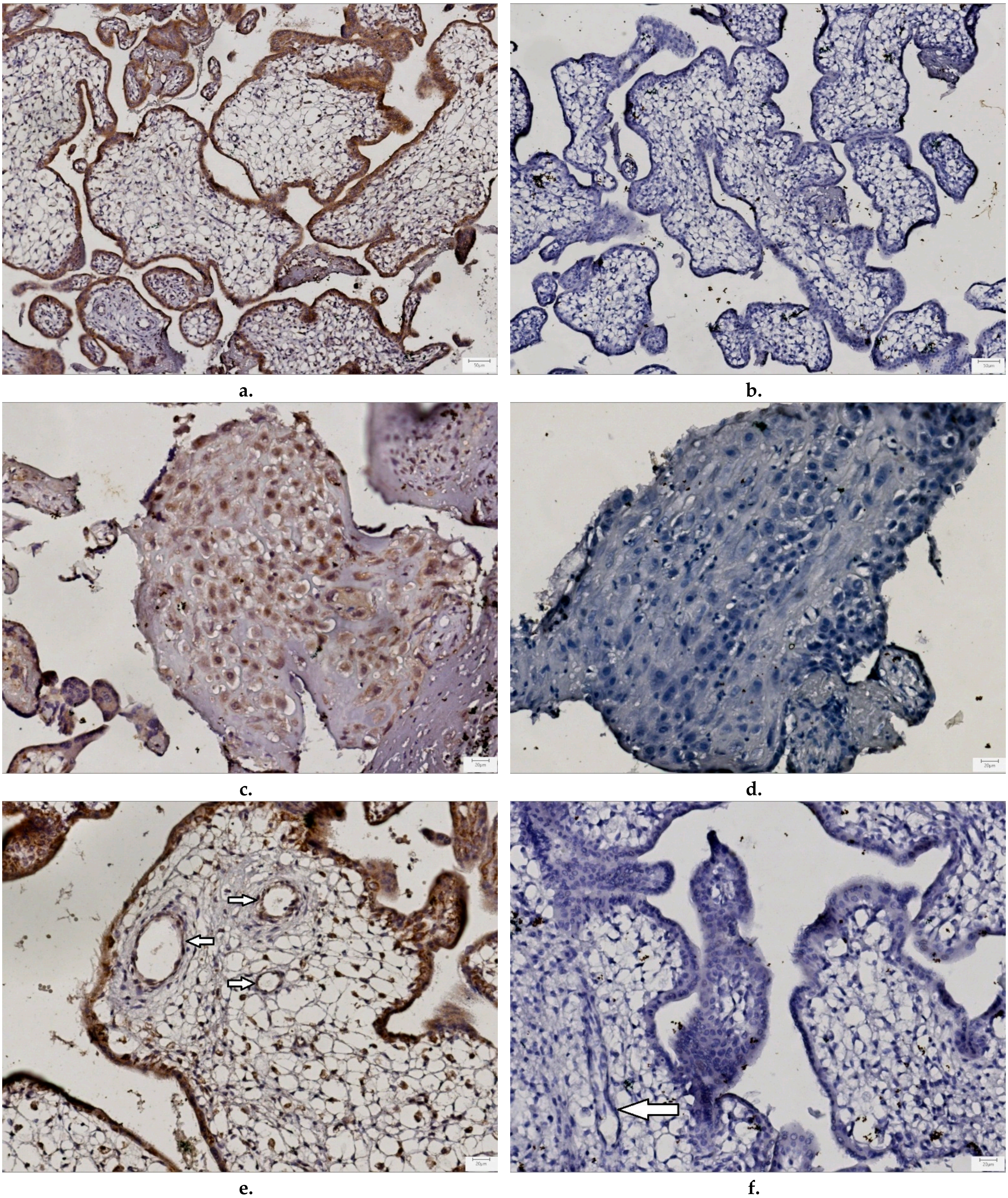

Figure S1. Representative micrographs displaying comparative positive immunostaining for PlGF (left panels) compared to immunohistochemical negative controls (right panels), at the level of: **a, b.** the syncytiotrophoblast (strong staining – left slide, magnification 10x for both), **c, d**. decidual cells (strong staining – left slide, magnification 20x for both), and **e, f.** villous endothelial cells (white arrows, moderate staining – left slide, magnification 20x for both).

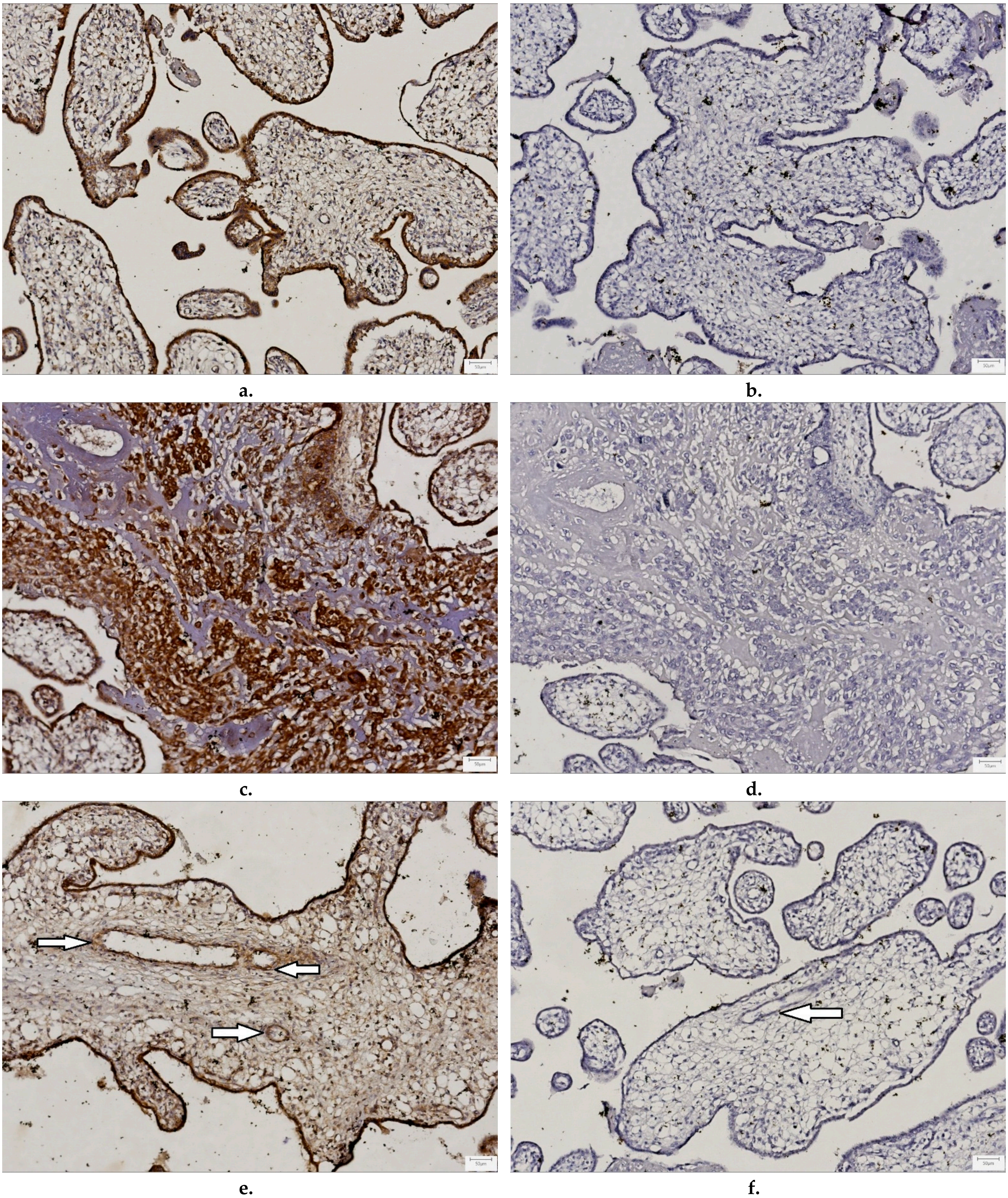

Figure S2. Representative micrographs displaying comparative positive immunostaining for VEGFR-1 (left panels) compared to immunohistochemical negative controls (right panels), at the level of: **a, b.** the syncytiotrophoblast (strong staining – left slide, magnification 10x for both), **c, d**. decidual cells (strong staining – left slide, magnification 10x for both), and **e, f.** villous endothelial cells (white arrows, moderate staining – left slide, magnification 10x for both).
